# LTR Retrotransposons in Fungi

**DOI:** 10.1371/journal.pone.0029425

**Published:** 2011-12-29

**Authors:** Anna Muszewska, Marta Hoffman-Sommer, Marcin Grynberg

**Affiliations:** Institute of Biochemistry and Biophysics, Polish Academy of Sciences, Warsaw, Poland; University of British Columbia, Canada

## Abstract

Transposable elements with long terminal direct repeats (LTR TEs) are one of the best studied groups of mobile elements. They are ubiquitous elements present in almost all eukaryotic genomes. Their number and state of conservation can be a highlight of genome dynamics. We searched all published fungal genomes for LTR-containing retrotransposons, including both complete, functional elements and remnant copies. We identified a total of over 66,000 elements, all of which belong to the Ty1/*Copia* or Ty3/*Gypsy* superfamilies. Most of the detected *Gypsy* elements represent *Chromoviridae*, i.e. they carry a chromodomain in the *pol* ORF. We analyzed our data from a genome-ecology perspective, looking at the abundance of various types of LTR TEs in individual genomes and at the highest-copy element from each genome. The TE content is very variable among the analyzed genomes. Some genomes are very scarce in LTR TEs (<50 elements), others demonstrate huge expansions (>8000 elements). The data shows that transposon expansions in fungi usually involve an increase both in the copy number of individual elements and in the number of element types. The majority of the highest-copy TEs from all genomes are Ty3/*Gypsy* transposons. Phylogenetic analysis of these elements suggests that TE expansions have appeared independently of each other, in distant genomes and at different taxonomical levels. We also analyzed the evolutionary relationships between protein domains encoded by the transposon *pol* ORF and we found that the protease is the fastest evolving domain whereas reverse transcriptase and RNase H evolve much slower and in correlation with each other.

## Introduction

Mobile elements are genome components that are able to move from one genetic locus to another. The best studied group of mobile elements are transposons (transposable elements) – widespread among all living organisms, they constitute a significant part of most analyzed genomes. In vertebrates and plants, transposon-derived content can exceed half of the whole genome [1]. Transposable elements have been shown to play a crucial role in genome shaping via recombination and expansion events, leading to chromosomal rearrangements and new gene neighborhoods [2], and they have also been shown to alter gene expression [3]. Examples are known where transposon fragments have gained new functions through exaptation and/or adaptation processes [4], [5].

Transposable elements (TEs) are traditionally divided into two major classes, based on their dispersion mechanisms [6]. Class I elements (retrotransposons) require an RNA intermediate in their transposition cycle. Retrotransposons synthesize a cDNA copy based on the RNA strand using a reverse transcriptase (RT) related to retroviral RT. Class II elements follow only an excision and insertion cycle. Their basic architecture is simpler, but many complex and variable DNA transposon types have been recently reported [7], [8]. Both classes encompass autonomous elements as well as non-autonomous elements which can be mobilized in *trans*, by exogenous enzymes.

**Figure 1:**
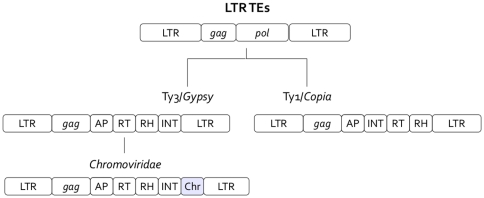
A schematic representation of LTR transposable elements present in fungal genomes.

Retrotransposons are further divided into five orders [9]: LTR TEs, characterized by long terminal direct repeats (LTRs) flanking the polyprotein genes, and four different non-LTR orders, named DIRS, PLE, LINE and SINE. Members of the LTR order usually encode two open reading frames (ORFs) (Figure 1). The first ORF is related to viral structural proteins, termed *gag*, and the latter, termed *pol*, is a polyprotein composed of an aspartic protease (AP) which cleaves the polyprotein, a reverse transcriptase (RT) which produces a cDNA copy of the transposon's RNA, an RNase H (RH) which splits the DNA-RNA hybrid and a DDE integrase (INT) which inserts the cDNA into the host's genome. DDE integrases are endonucleases with a DDE motif and are distantly related to *Mariner* DNA DDE transposases [10]. The order of domains encoded in the *pol* ORF varies between different LTR retrotransposon families, and often additional domains are inserted, e.g. chromodomains [11], [12].

LTR retrotransposons are classified into five superfamilies (Figure 1): Ty1/*Copia* (*Pseudoviridae*), Ty3/*Gypsy* (*Metaviridae*), Bel/Pao, retroviruses and ERV (endogenous retroviruses). Up to now, members of the three last superfamilies have been detected only in metazoan hosts, while Ty1/*Copia* and Ty3/*Gypsy* elements have been reported in all eukaryotic lineages. In filamentous fungi, both Ty3/*Gypsy* and Ty1/*Copia* elements have been detected, with *Gypsy* being the most abundant [13]. Most fungal transposons of the Ty3/*Gypsy* superfamily are classified as *Chromoviridae* because of the presence of a chromointegrase (an integrase with a C-terminal chromodomain) [14]. Chromoviruses have been detected in almost all Eukaryotic lineages [15].

As LTR retrotransposons require a multi-compound machinery to be mobile, they easily become non-autonomous. Often one genome harbors both an autonomous element and a related non-autonomous element which acts like a parasite of the functional copy [16].

Genomes tend to fight against the expansion of transposable elements and filamentous fungi have become specialists in this field: at least three of the main transposon silencing mechanisms –repeat-induced point mutation (RIP), methylation and quelling, as well as the recently discovered sex-induced silencing (SIS) mechanism – have been described in fungi [17], [18], [19], [20].

The presence of transposable elements in fungi has been first reported in yeast in the 1970s, but LTR TEs in filamentous fungi were discovered more than 10 years later [21], [22]. Although some LTR retroelements have been proven to be functional (e.g. the MAGGY element from *Magnaporthe grisea* [23]), most of the detected mobile elements harbor many stop codons in coding regions which prevents them from being functional.

There has been no whole kingdom analysis of LTR TEs in fungi yet; only single genomes, such as those of *Neurospora crassa* or *M. grisea*, have been completely scanned for LTR retroelements. The *M. grisea* genome study has shown an uneven distribution of mobile elements along chromosomes and the presence of novel elements [24]. Here we present the results of a large-scale search for LTR TEs in 59 fungal genome sequences. We searched for both full-length and truncated elements and we tried to assign all of them to one of the known LTR TE superfamilies. We attempted to analyze our data from a genome-ecology perspective, i.e. viewing the genome as an environment for (selfish) mobile elements. This approach leads us to questions such as: do high LTR TE counts in fungi result typically from expansions of single clones (many copies of one transposon), single families (one LTR TE type dominates a particular genome), or from a high abundance of all types of elements? Are certain types of genomes (e.g. representing a certain taxonomic group) better environments for mobile elements? Does the lifestyle of the organism correlate somehow with its LTR TE content? Does the presence or absence of known genome defense mechanisms correlate with TE abundance or profile?

In higher eukaryotes the TE content has been shown to be directly related to the effective population size of the host organism [25]. Studies in *Oxytricha trifallax* have proven that TEs can influence the adaptive capabilities of cells [26]. Our preliminary analysis in fungi shows a great diversity in the abundance of LTR TEs among the analyzed genomes, also between closely related species. At the same time, the variability of the identified elements is not that strong: only two LTR TE families (Ty1/*Copia* and Ty3/*Gypsy*) are represented in fungi, and the majority of the detected Ty3/*Gypsy* representatives belong to *Chromoviridae*. An analysis of the most successful element in each genome identifies *Chromoviridae* as the dominating group in fungal genomes. Our data shows that transposon expansions in fungi usually involve both an increase in copy number of individual elements and an increase in the number of different elements.

## Results

### Identification of LTR transposable elements in fungal genome sequences

**Figure 2:**
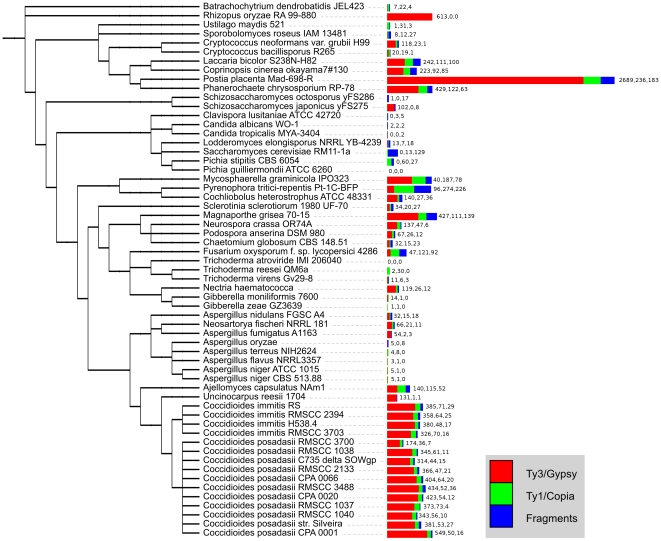
The number of LTR transposable elements per genome in the dataset. The length of the bar is proportional to the number of detected LTR retrotransposons of the indicated types; the numbers are also depicted next to every bar. The tree image was prepared with iTol.

LTR retrotransposons were detected in 58 out of the 59 analyzed fungal genomes – only *Trichoderma atroviride* lacks any LTR elements. Because most tools designed for the detection of mobile elements have been developed for genomes other than fungal, multiple programs have been applied in this study. Two programs dedicated to LTR retrotransposons were used: LTR Finder and LTR harvest, and one universal tool: a combination of RepeatModeler and RepeatMasker. Both full-length elements and remnant copies were considered in the statistical analysis, as our goal was to explore evolutionary tendencies, not only the current abundance of functional LTR retrotransposons. However, the presence of integrase, transposase, aspartic protease or RNase H was considered a sign of recent activity. More than 66,500 representatives have been identified, the majority of them being short and truncated (see Table S1; for raw data see Table S2). ORF searches revealed that 16,289 elements still carry at least one of the analyzed proteins (Gag, INT, RT, AP or RH) and are therefore considered functional. Genomes abundant in intact elements are also rich in remnant copies. Figure 2 shows the number of elements from each superfamily that are probably still functional per genome. The average LTR retrotransposon number is 1129 per genome and the median is 796. The median is a better measure of the most often encountered state for data with uneven distribution. LTR retrotransposons carrying at least one of the above listed ORFs appear with an average of 276 per genome and with a median of 133. In the following analysis we concentrate on these potentially functional elements.

As stated before [13], the best studied genomes of filamentous fungi are poor in mobile elements. Ascomycota, the best sampled phylum in our study (49 genomes), have genomes with a very variable number of LTR retrotransposons. Most model fungi, belonging to Eurotiales and Saccharomycetes, have very low numbers of LTR retrotransposons: 65 for *Aspergillus nidulans*, 21 for *Saccharomyces cerevisiae*. These species are not representative for the whole phylum. In other ascomycetous groups, such as Dothideomycetes (*Mycosphaerella graminicola* (605)) and Onygenales (*Ajellomyces capsulatus* (307), *Coccidioides immitis* (412–485), *C. posadasii* (217–615)), many genomes are abundant in potentially functional mobile elements. It should be mentioned here that the large differences in LTR TE abundance between different strains of the two analyzed *Coccidioides* species may result from differences in coverage of the genome sequences, since the number of identified elements correlates well with the length of the genomic sequence assemblies (data not shown). Sordariales genomes, with the model fungi *Neurospora crassa* (190) and *Podospora anserina* (105), and with the opportunistic *Chaetomium globosum* (70), are not abundant in LTR retroelements, consistent with the well studied mechanisms for genome defense against duplicated content which operate in these organisms [17], [27], [28]. Hypocreales, represented by Trichoderma (0–35 elements) and Nectriaceae (Giberella 2–15, *Nectria haematococca* 157 LTR TEs), have genomes with moderate and low LTR retrotransposon content. *Magnaporthe grisea*, the only sequenced Magnaporthales representative, outnumbers all other analyzed Sordariaceae, showing a very high LTR retrotransposon content (677 elements) which corroborates results reported in the genome publication [24]. The pattern of LTR retrotransposon distribution in Ascomycota is not clear; some orders have diverse representatives, like Onygenales: the saprophytic *Uncinocarpus reesei* has a low LTR retrotransposon number (133) whereas the systemic infection-causing species have an elevated LTR retrotransposon content (Coccidioides 217–615, *Ajellomyces capsulatus* 307). On the other hand, genomes classified to the subphyla Taphrinomycotina and Saccharomycotina have LTR retrotransposon numbers below the average for the whole phylum.

The second best represented phylum in our dataset is Basidiomycota, with 8 genomes. Interestingly, both measures for the average value of LTR retrotransposon content per genome, the mean 607 (1980 remnants) and the median 278 (961 remnants), are higher than for Ascomycota (mean 218 (969 remnants), median 119 (796 remnants)). However, the distribution is far from even. Four genomes (*Postia placenta* (3108), *Laccaria bicolor* (453), *Coprinopsis cinerea* (400), *Phanerochaete chrysosporium* (614)) have strong mobile element expansions causing whole-genome size growth while the rest (*Ustilago maydis* (35), *Sporobolomyces roseus* (47), *Cryptococcus neoformans grubii* (155), *Cryptococcus bacillisporus* (40)) are scarce in LTR retrotransposon content. Plant-related Basidiomycota (both symbiotic and pathogenic: *P. placenta*, *L. bicolor*, *P. chrysosporium*) encode multiple LTR retrotransposons, with the exception of *U. maydi*s which has a very compact genome [29]. The tiny genome of *Sporobolomyces roseus* also has a modest LTR TE number.

*Postia placenta* represents an interesting case. This species exhibits a huge expansion of LTR TEs, outnumbering all other known fungi. The elements identified in the *P. placenta* genome can be grouped into 262 LTR TE families (where a family is characterized by more than 80% similarity over the whole DNA sequence). The increase in overall LTR TE number in *P. placenta* must have been achieved by the multiplication and variation of many groups of elements, as the closest relative in the analyzed genome set, *Phanerochaete chrysosporium*, has representatives of only 97 families. The abundance of LTR TE in *P. chrysosporium* genome was previously reported [30]. Many of the elements identified in the *P. placenta* genome encode at least one protein domain and thus should have expanded recently, which might indicate a recent stress in the history of this species and/or a recent decrease in the effective population size [25]. This fungus is unique among cellulose degrading microbes in its glycoside hydrolase gene set and reveals many unusual biochemical features [31]. It would be tentative to study the influence of such an outburst of retrotransposons on the adaptation of *P. placenta* to its ecological niche.

The only included representative of early diverged Eumycota – *Batrachochytrium dendrobatidis*, belonging to the phylum Chytrydiomycota – has a low number of LTR elements (33). This is in contrast with the only Mucoromycotina in this study, *Rhizopus oryzae*, which encodes as many as 742 LTR retrotransposons [32].

Since we have not found any taxonomic pattern in genomic LTR TE abundance, we looked whether there was any correlation between the number of LTR TEs per genome and the organism's lifestyle or ecological niche (see Table S1). We observed several tendencies. First, genomes expand in plant related fungi. Second, in Ascomycota saprophytes are generally less abundant in LTR TEs than in non-saprophytes (Table S1). Since our findings are quite weak, we conclude that other factors, not included in our analysis, may influence LTR TE abundance in fungi.

### Evolutionary analysis of reverse transcriptase (RT), RNase H (RH), integrase (INT) and aspartic protease (AP)

**Figure 3:**
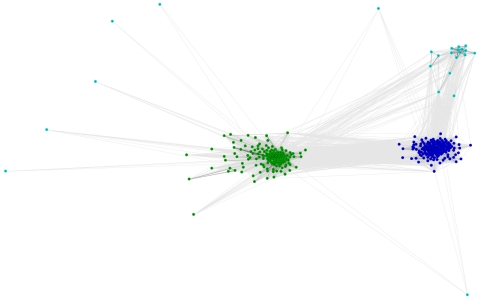
CLANS clustering of RT domains. Dark blue – Ty3/*Gypsy*, green – Ty1/*Copia*, light blue – fragmented or complex transposons. The intensity of the connecting lines reflects the level of sequence similarity.

**Figure 4:**
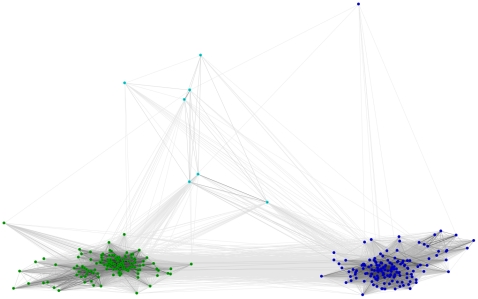
CLANS clustering of RH domains. Dark blue – Ty3/*Gypsy*, green – Ty1/*Copia*, light blue – fragmented or complex transposons.

**Figure 5:**
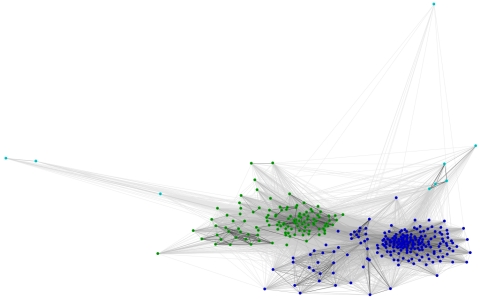
CLANS clustering of INT domains. Dark blue – Ty3/*Gypsy*, green – Ty1/*Copia*, light blue – fragmented or complex transposons.

We then tried to investigate the evolutionary history that led to the currently observed LTR TE profiles. We estimated the divergence of LTR retrotransposons basing on sequence similarity and localization of protein domains within the transposon sequence. For this purpose we used several domains that are the milestones of phylogenetic studies: RT, RH, INT and AP. For each of these domains, CD-HIT and CLANS clustering were performed. The first step reduced the number of analyzed sequences filtering out the highly similar variants of each element. The second step produced a graphical overview of the variability of the protein domain of interest. In all cases three clades could be identified, corresponding to Ty3/*Gypsy* (with the dominance of *Chromoviridae* elements, but including also some representatives with similarity to the Ylt1 element from *Yarrowia lipolytica* [33]; shown in Figures 3, 4, 5 in dark blue), Ty1/*Copia* (green), and an outlier group (shown in light blue; discussed below). As expected, the closer to each other the domains are located within the *pol* ORF, the more similar the clustering images are. The clustering images of reverse transcriptase and RNase H are almost identical, corroborating the opinion that both domains are indispensable for full catalytic functionality of LTR elements. The RT and RH domains are transmitted together and form one protein when expressed [34].

### Reverse transcriptase

The reverse transcriptase (RNA polymerase) is responsible for DNA synthesis using RNA as a template. The RT domain in the Pfam database is represented by two separate profiles: RVT_1 (Pfam:PF00078) and RVT_2 (Pfam:PF07727). Both are described as present in a variety of mobile elements. In our sequence searches the RVT_1 profile associated with Ty3/*Gypsy*, whereas RVT_2 associated with Ty1/*Copia* LTR retrotransposons. Three groups are clearly distinguishable in the CLANS clustering image (Figure 3). Ty3/*Gypsy* form the biggest clade visible on the right side of the image (dark blue). Most filamentous fungi carry more Ty3/*Gypsy* than Ty1/*Copia* elements. In the left part of the image Ty1/*Copia* elements are grouped. Both Ty3/*Gypsy* and Ty1/*Copia* elements were identified in genomes of all taxonomic groups in the analyzed dataset. Some sequences diverge from these major clans, forming a third group (light blue). This group is comprised of degenerated sequences scattered with stop codons and of complex transposons which result from multiple insertions into one genomic locus. These complex transposons contain many additional domains inserted into the structure of typical Ty1 or Ty3 elements (to illustrate this phenomenon, we present the domain architecture of 4 such complex transposons in Figure S1).

### RNase H

In retrotransposons RNase H is responsible for the degradation of the RNA template in the DNA-RNA hybrid. The Pfam RNaseH profile (PF00075) is not sensitive enough to detect transposon-related RHs. Sequence searches conducted with this profile rendered less than 10% of RH domains present in the analyzed dataset and in reference sequences known from literature. This phenomenon can be explained by the divergence of RH domains in LTR retrotransposons from the canonical RNase H described in *E. coli* and viruses [34]. In order to find specific RHs a new “LTR-TE-oriented” profile was built. For this purpose, sequences of the *pol* polyprotein of fungal LTR retrotransposons known from literature were collected from the NCBI protein database. Using this profile, we found three separate clans. The obtained CLANS image (Figure 4) is almost identical to that for the RT clustering, corroborating their direct neighborhood in the mature reverse transcriptase machinery.

### Integrase

The integrase catalyzes insertion of the retrotransposon cDNA into the genome of a host cell. It consists of three protease-resistant domains, but only rve (PF00665) – the integrase core domain – is well conserved among a variety of organisms. It belongs to the Pfam RNase H clan. This domain was extracted and clustered. The result shows two major clades as well as several outlier sequences (Figure 5). The differences between Ty3/*Gypsy* and Ty1/*Copia* are less sharp here than in the images for RT and RH, resulting in a more compact relationship between the clusters. The outlying sequences visible in the CLANS output are in most cases non-functional copies, with many stop codon mutations. The integrase domain is localized differently in the *pol* gene in Ty3/*Gypsy* and Ty1/*Copia* retrotransposons. In Ty3/*Gypsy* elements integrase is usually the last element in the *pol* gene whereas in Ty1/*Copia* it is located between the sequences coding the AP and the RT. The clustering analysis shows also that integrases are less diverged between Ty3/*Gypsy* and Ty1/*Copia* than the RT and RH domains.

### Aspartic protease

**Figure 6:**
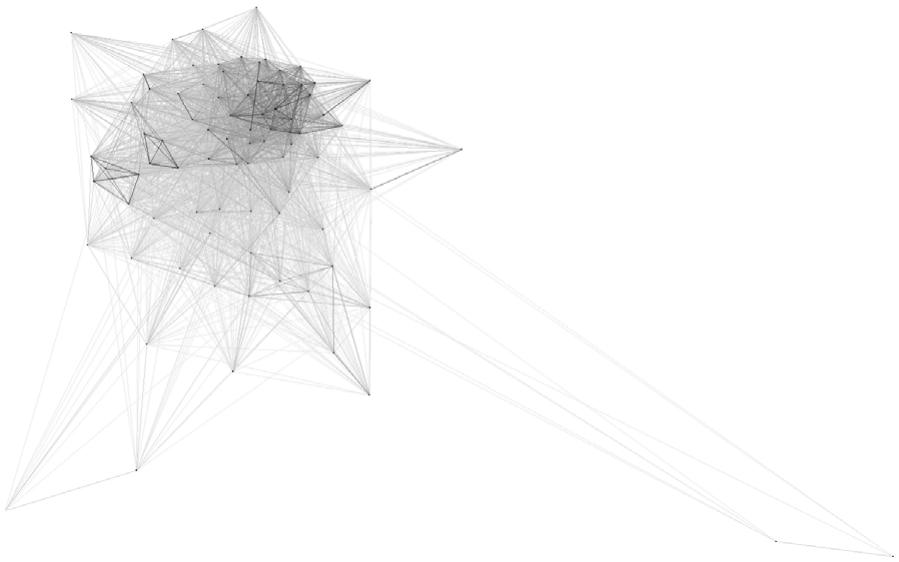
CLANS clustering of AP domains. These sequences do not form any well-defined groups.

The aspartic protease is responsible for processing the large transposon transcripts into smaller protein products. Four different aspartic protease profiles from the Peptidase_AA clan (CL0129) were used in our study. Two of them are dominant and unevenly distributed: RVP (PF00077) is found in Ty1/*Copia* and RVP_2 (PF08284) in Ty3/*Gypsy* LTR TEs. The Peptidase_A2B (PF12384) and Peptidase_A2E (PF12382) domains are found only in several copies. The high variability of the AP resulted in a very low detection rate in LTR TE sequences when compared to the RT, RH and INT domains. There are neither clades specific for fungal taxonomic groups nor clades related to a specific type of LTR TE (Figure 6). The aspartic proteases are said to be difficult to analyze due to their low similarity and different evolution rates. This subject is being studied in detail by the authors of GyDb [35].

Taken together, the presented CLANS results show that all analyzed *pol*-encoded domains display a similar clustering pattern – with Ty1/*Copia* and Ty3/*Gypsy* elements forming two major clusters – which remains in concordance with the currently accepted view of LTR TE evolutionary history.

### Most successful element analysis

In order to see which kinds of transposable elements are most successful in which genomes, we searched for the highest copy number LTR TE in every analyzed genome. The identified elements were then subjected to a phylogenetic analysis. The *pol* gene (encoding the RT, RH, INT and AP proteins) was chosen for this analysis because of its conservation. The only other ORF present in most LTR retrotransposons – *gag* – is much more variable and it is difficult to identify using sequence profiles. Not all of the most successful elements encode a complete set of *pol*-encoded protein domains suggesting their non-autonomous character or a non-functional state. This distribution depicts the battle between genome defense mechanisms and the expansion of mobile elements in fungi. The presence of multiple stop codons in the majority of the characterized mobile elements – which most likely renders them non-functional – can be regarded as an emanation of this phenomenon. Most of the elements have incomplete *pol* genes lacking one or more of the core components (INT, RT, RH, AP).

Some most successful elements have been excluded from the phylogenetic analysis: the biggest LTR TE families from *Gibberella zeae*, *Candida tropicalis* MYA-3404, *Candida albicans* WO1, *Schizosaccharomyces octosporus* yFS286, *Aspergillus flavus* and both *Aspergillus niger* genomes have only one member and in consequence cannot be considered successful in expanding along the genome, *Candida guilliermondii* and *Trichoderma atroviride* have no LTR TE with a detectable *pol* gene at all. Some of these genomes have already been described as scarce in repetitive content [36], [37].

**Figure 7:**
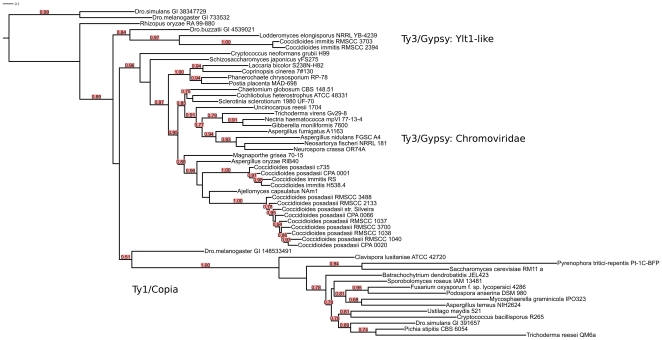
Maximum-likelihood phylogenetic tree of the most successful LTR transposable elements in the analyzed fungi. Concatenated amino acid sequences of RT, INT, RH and AP protein domains were used as the dataset. The phylogenetic analysis was performed with PhyML. Approximate likelihood ratio test SH-like branch supports above 50% are shown. The tree image was prepared with iTol.

The obtained phylogenetic tree (Figure 7) depicts the relationships between the most successful elements from all analyzed genomes. It shows clearly that *Chromoviridae* (belonging to the Ty3/*Gypsy* superfamily) are almost twice as successful as Ty1/*Copia* in dominating fungal genomes. Elements similar to *Yarrowia lipolytica* Ylt1 dominate in 3 genomes.

In almost every taxonomical range the history of genome invasion was different which is reflected by the differences and conservation of the most successful elements among taxa. The genomes of *Coccidioides immitis* and *C. posadasii* were invaded by at least two very dynamically dispersing elements, all belonging to Ty3/*Gypsy*. Two *C. immitis* strains have Ylt1-like elements related to those in the *Lodderomyces elongisporus* genome. The *Chromoviridae* elements dominating in most Coccidioides and in the *Ajellomyces capsulatus* genome are closely related. Four different Agaricomycotina genomes were conquered by a very similar element. Ty3/*Gypsy* elements insert preferentially into heterochromatin regions whereas Ty1/*Copia* retrotransposons have been reported to integrate into transcriptionally active regions of the genome [14]. Recently, heterochromatin has been considered a crucial player in speciation [38]. If so, the role of abundant TE elements in this process needs elucidation. The activity of mobile elements could serve as an indicator of a strong need for adaptation to new environmental conditions.

The phylogenetic tree shown in Figure 7 does not overlap with the taxonomic classification of the host genomes, indicating that there are no general rules as to which types of LTR TEs are most successful in a particular taxon. In the Ascomycota division the frequency of TEs differs between subphyla (Saccharomycotina, Taphrinomycotina and Eurotiomycotina). *Saccharomyces cerevisiae* has more *Copia* elements than *Gypsy*, and in our study also the most successful element in this species is a Ty1/*Copia* type element, which is consistent with previous studies [39]. Some elements seem to be common to a taxonomical group, e.g. the most successful Ty3/*Gypsy* element in three Hypocreales species: *Nectria haematococca*, *Gibberella moniliformis* and *Trichoderma virens*. In the same order, a *Copia* element was the most successful in the genome of *T. reesei*. The highest-copy elements in most Eurotiales (*Aspergillus nidulans*, *Neosartorya fischeri* and *A. fumigatus*) display similarity to that of *Neurospora crassa*, a member of Sordariales.

Basidiomycota genomes were ‘invaded’ by different elements. The most successful elements in two Cryptococcus genomes belong to two separate LTR TE superfamilies. Surprisingly, four other Basidiomycota genomes: *Phanerochaete chrysosporium, Coprinopsis cinerea, Laccaria bicolor* and *Postia placenta*, were invaded by Ty3/*Gypsy* elements which cluster together on the phylogenetic tree. These elements might descend from some common ancestor.

The most successful element in the genome of the orphan representative of Mucoraceae, *Rhizopus oryzae*, is a Ty3/*Gypsy* LTR TE. This element does not cluster together with any other sequence, possibly as a consequence of its early divergence. The only chytrid *Batrachochytrium dendrobatidis* was invaded by a Ty1/*Copia*.

## Discussion

LTR TEs are ubiquitous elements present in almost all analyzed genomes. A large fraction of the identified LTR TEs is degraded but full copies can also be found. LTR TE variability can be observed even between very closely related species (e.g. between the two *Coccidioides* species), showing that otherwise highly similar organisms can be in some cases identified by their LTR TE content, whereas in other cases metabolically distant strains can harbour almost identical (presumably ancestral) LTR TEs (*Aspergillus niger*). These two examples suggest that using LTR TEs for strain differentiation could be useful for analyzing recent changes, although it is clearly not suitable for the analysis of old historical events. Also, gradual changes in LTR TE content should be considered for highly “transpositionally” active and dynamically changing genomes; even strains of a single species can display a certain percentage of variability in their mobilome during cultivation, as an answer to different environmental conditions.

In this work we searched only for LTR-containing retrotransposons and we were able to identify elements representing both of the LTR TE superfamilies known to reside in fungal genomes: Ty3/*Gypsy* and Ty1/*Copia*. A preference for *Copia* over *Gypsy* elements can be noticed in some fungi (e.g. *Saccharomyces cerevisiae*) but these are exceptions – in most genomes both groups are present in high numbers and *Gypsy* elements prevail. Of the Ty3/*Gypsy* superfamily, *Chromoviridae* representatives were most frequently found in the analyzed genomes. We observe that in genomes rich in LTR TEs the highest copy element is usually more numerous than in genomes scarce in LTR TEs.

More sequencing data will be necessary for general patterns in the distribution of LTR transposons in fungal genomes to become visible. Of the Ascomycota, *Ajellomyces capsulatus* and *Coccidioides* – both representatives of pathogenic orders classified to Onygenales – have many LTR TEs while *Uncinocarpus reesii* – a closely related non-pathogen – has very few elements. Also among the Pezizomycotina, the non-pathogenic *Neurospora crassa* shows a low TE content whereas the plant pathogen *Magnaporthe grisea* displays a high TE content. However, the simplifying conclusion that the presence of multiple LTR TEs is directly related to a pathogenic niche should not be drawn on the basis of these scarce examples.

Basidiomycota are strongly underrepresented in our dataset, which prevents us from identifying any patterns of LTR TE distribution in this taxon. More genomes of Basidiomycota are currently being sequenced so a broader analysis will be possible in the near future.

Among the basal fungi, *Rhizopus oryzae*, representing Mucoraceae in our study, has a genome abundant in LTR TEs, but another basal fungus, *Batrachochytrium dendrobatidis* – the only available Chytrydiomycota – has a completely different TE profile, with multiple remnant copies and few complete elements. It has been suggested that basal fungi should have genomes more abundant in Chromoviruses (the most numerous class of LTR TE in Fungi) than Asco- and Basidiomycota [15]. One of the possible hypotheses is that *B. dendrobatidis* is an exception among basal fungi in that it carries a very efficient TE elimination system. If so, other basal fungi would still be rich in TEs. The alternative scenario is that the TE content of most Chytrydiomycota is more similar to that of the common Metazoa/Fungi ancestors. The typical fungal TE repertoire would then be established later, in separate evolutionary events. TE loss (compared to *Rhizopus oryzae*) could have been favored later in the evolution of *B. dendrobatidis*, similarly as can be observed in the genomes of the Eurotiales as well as of certain Sordariales, Taphrinomycotina and Saccharomycotina. More sequencing data regarding basal fungal lineages would be necessary to decide which of these two scenarios has actually taken place.

TE expansions are popular, but seem to appear independently in distant genomes at different taxonomical levels. Some expansions seem to have occurred in the ancestor of Onygenales (*Coccidioides*, *Ajellomyces*) and in the ancestor of Polyporales (*Phanerochaete*, *Postia*). Others are shared only by strains of closely related species. Most expansions are genome specific and may constitute a response to some specific conditions favoring genome variability instead of stability. Retrotransposon expansions seem to be individual stories in *Coccidioides*, where highly divergent LTR TEs dominate the genomes of closely related strains, in contrast to *Aspergillus niger*, where the TE content is common. In *Coccidioides*, high variability might have been advantageous because of the extreme changes in their ecological niches and the recent speciation between *C. immitis* and *C. posadasii* As mentioned before, TE content in *Coccidioides* may be related to genome sequencing coverage, not to real numbers of TEs.

In our analysis we could follow the stepwise process of degradation of an LTR TE, with the least conserved domains being proteases and *gag*'s (according to protein definitions and profiles supplied by Pfam). The core integrase domain (rve) is one of the top scoring domains in Pfam showing that this core fold is favored in evolution. We show that the integrase domain is conserved in transposable elements; however, this observation is directly dependent on the Pfam domain definition. *gag* genes are said to be diversified and the available protein sequence profiles are not applicable for distant elements.

The INT-RH-RT set seems to be inherited together, while the protease (AP) is more variable. This could mean that the protease can be exchanged for some similar “module”, but it is more likely that the protein domain profiles of the various aspartic proteases available in databases are still not sensible enough to detect all the existing variants of this protein.

The obtained data points to a complex phylogeny of LTR TEs in fungi, including expansions, losses and transfers of retrotransposons in almost every species and strain. LTR TE number, distribution and state of conservation can be a valuable source of information about genome dynamics and evolutionary strategies. Further, we still think that there may be a dependency between the quantity and quality of LTR retrotransposons and the host ecological niche, but more sequencing data would be required to investigate this possibility.

## Materials and Methods

### Data Mining

Genome sequences have been obtained from sequencing consortia: Fungal Genome Initiative (BROAD Institute) and the DOE Joint Genome Institute (JGI). All downloads were performed before December 27^th^, 2008, and only genomes publicly available in March 2010 were included into the analysis. To detect all the available sequences corresponding to LTR retrotransposons, three different programs were applied on each genome sequence. There are no fungal-specific transposable element-directed tools thus a combined search was needed to obtain reliable results. Two tools, LTR harvest [40] and LTR Finder [41], are dedicated to this group of mobile elements. Both programs search for LTR TE-specific features like long terminal repeats. The third tool we used is a set composed of RepeatModeler followed by RepeatMasker 3.3.0. RepeatMasker was run with the RepBase library of manually curated mobile element and repetitive DNA sequences as a reference dataset [42]. RepeatModeler was run separately on each genome in order to produce consensus sequences for genome specific repeat classes. All genomic consensus sequences together with the fungal subset of RepBase database were used as the reference library for RepeatMasker searches, carried out on each genome. Results from all three predictions were merged, yielding a set of full length and truncated LTR retrotransposons. Duplicated hits were removed from the cumulative result based on overlaps exceeding 80% of length of the shorter sequence.

### Most successful element analysis

Records for each genome were clustered with a threshold of 80% of nucleotide sequence similarity using cd-hit-est from the CD-HIT package in order to get LTR retrotransposon family count per genome [43]. Genomes harboring only orphan elements were discarded form this analysis. The richest family from each genome was selected and the longest representative from this family was further analyzed. Each element was translated in 6 frames and the coding sequence was extracted. If the longest element had the coding region distributed among many frames or was a complex transposon, the next longest element was chosen. All elements were clustered with CLANS to observe general tendencies. The sequences corresponding to 4 protein domains: RT, INT, AP and RH, were extracted. Protein sequences were aligned with the localpair iterative algorithm implemented in Mafft [44]. Conserved columns from each multiple sequence alignment were chosen with TrimAl [45]. The selected set of columns was concatenated with an in house Python script. The most suitable model for phylogenetic analysis was selected with ProtTest [46]. According to the AIC criterion, an LG+G+F model with a score of 0.63 was the most suitable for the dataset. Maximum likelihood analysis of the pol region was carried out with PhyML on-line facility [47] with the following settings: LG model of amino acid substitution, 4 categories in gamma model with the shape parameter estimated as 2.48. Instead of bootstrap replicates branch support was calculated using an approximate likelihood ratio test. Trees were visualized in iTOL [48]. *Drosophila simulans* (GI: 38347729) and *D. melanogaster* (GI: 733532) sequences belonging to Bel/Pao LTR retrotransposons were set as an outgroup. *D. simulans* (GI: 391645) was used as a type representative of *Copia* elements. *D. buzzatii* (GI:4539021) *Osvaldo* transposon was used as a *Gypsy* outgroup and *D. melanogaster* (GI:148553491) as a *Gypsy* type specimen.

### Protein analysis

Since the order of the encoded proteins and the sequence similarity of the reverse transcriptase form the base for LTR retrotransposon classification, repetitive elements were screened for retrotransposon-related protein domains. Each putative LTR retrotransposon was translated in 6 frames with transeq from the EMBOSS package [49] and searched with HMMsearch (from HMMer3.0 package [50]) against 13 HMM (Hidden Markov Model) profiles corresponding to 8 different protein domains (INT, RT, AP, RNase H, Gag, Chromo, and two RT-related: RVT_thumb and RVT_connect) included in the Pfam25 database [51] and one developed specially for this project: the Pfam RNase H profile does not include retrotransposon RNase H sequences which are known to vary from canonical RNase H sequences [34], so we made our own RNase H profile, using known fungal retrotransposon RNase H sequences together with two PDB [52] non-fungal structures for alignment guiding purposes. Sequence searches were automated with pfam_scan.pl, a tool available at the Pfam database site [51], which enables the user to create a database of HMM profiles to be searched with a database of protein sequences. Protein sequences corresponding to the mentioned protein domains were clustered together with CD-HIT to remove highly similar sequences. The clan representatives were clustered in CLANS, a tool which enables clustering and visualization of sequence similarities [53].

## Supporting Information

Figure S1 Domain architecture of four selected complex transposons. 1 and 2 are retrotransposons found in the *Pyrenophora tritici-repentis* genome, 3 was identified in *Rhizopus oryzae* and 4 in the *Chaetomium globosum* genome.
(TIF)

Table S1 Transposon content and selected features of all analyzed fungi. Includes citations to references [54]–[88].
(XLS)

Table S2 All mobile elements identified in this work (raw data).
(CSV)
