# Supplementary figures and images for: LTR Retrotransposons in Fungi
Source: PLoS One. 2011 Dec 29;6(12):e29425. doi: 10.1371/journal.pone.0029425 (PMC3248453; doi:10.1371/journal.pone.0029425)

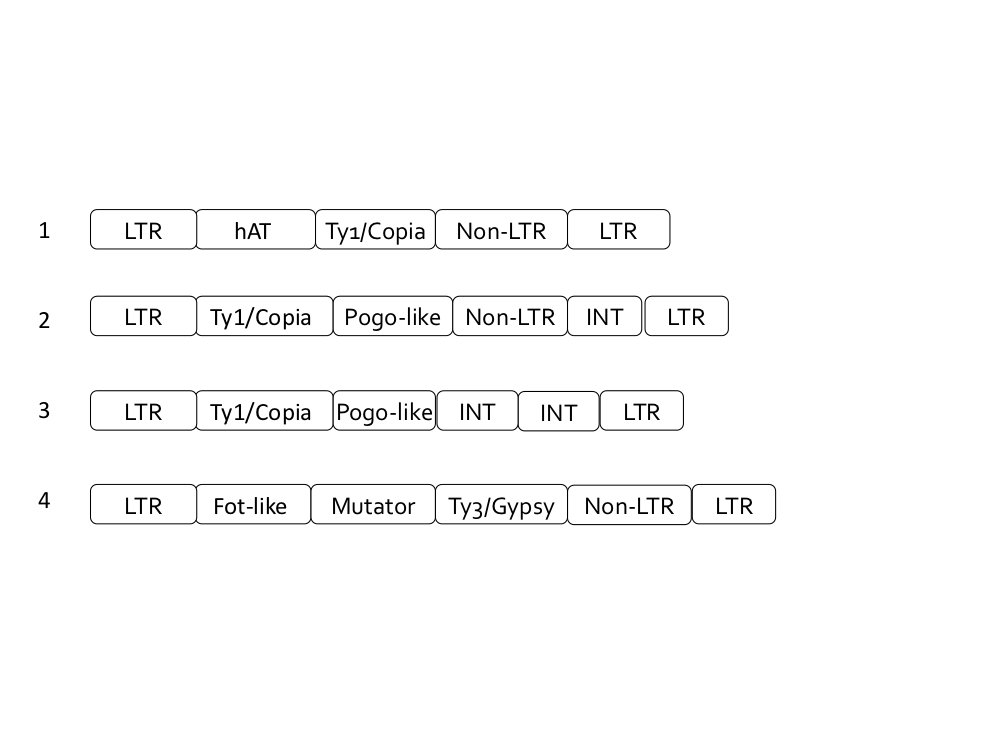

Supplement: Figure S1 — Domain architecture of four selected complex transposons. 1 and 2 are retrotransposons found in the Pyrenophora tritici-repentis genome, 3 was identified in Rhizopus oryzae and 4 in the Chaetomium globosum genome. (TIF) [file pone.0029425.s001.tif]
